# Unmet Needs and Their Impact on Quality of Life and Symptoms in Myelodysplastic Neoplasm Patients and Caregivers

**DOI:** 10.3390/cancers17091587

**Published:** 2025-05-07

**Authors:** Elena Crisà, Daniela Cilloni, Marta Riva, Enrico Balleari, Daniela Barraco, Beatrice Manghisi, Lorenza Borin, Michela Calmasini, Anna Calvisi, Isabella Capodanno, Matteo Giovanni Della Porta, Elisa Diral, Bruno Fattizzo, Susanna Fenu, Stefania Paolini, Carlo Finelli, Claudio Fozza, Chiara Frairia, Valentina Giai, Mauro Turrini, Maria Antonia Isoni, Federico Itri, Luca Maurillo, Alfredo Molteni, Giuseppe Alberto Palumbo, Anna Maria Pelizzari, Federica Pilo, Antonella Poloni, Costanza Bosi, Grazia Sanpaolo, Rosaria Sancetta, Cristina Amato, Valeria Santini, Maria Teresa Voso, Sam Salek, Tatyana Ionova, Annamaria Nosari, Esther Natalie Oliva

**Affiliations:** 1 Candiolo Cancer Institute, FPO-IRCCS, 10060 Candiolo, Italy; 2 A.O. Ordine Mauriziano di Torino, Department of Clinical and Biological Sciences, University of Turin, 10128 Turin, Italy; daniela.cilloni@unito.it; 3 Hematology, ASST Grande Ospedale Metropolitano Niguarda, 20162 Milan, Italy; marta.riva@ospedaleniguarda.it; 4 IRCCS Policlinico Ospedale San Martino, 16132 Genoa, Italy; eballeari@gmail.com; 5 Dipartimento di Ematologia, ASST Sette Laghi, Ospedale di Circolo e Fondazione Macchi, 21100 Varese, Italy; daniela.barraco@asst-settelaghi.it; 6 Hematology, Ospedale San Gerardo, 20900 Monza, Italy; beatrice.manghisi@irccs-sangerardo.it (B.M.); lorenzamaria.borin@irccs-sangerardo.it (L.B.); 7 Associazione Italiana Pazienti Sindromi Mielodisplastica AIPASIM, 20162 Milan, Italy; calmasinim@gmail.com (M.C.); annamaria.nosari@gmail.com (A.N.); 8 SC Ematologia, CTMO e Laboratorio Specialistico, Ospedale S. Francesco, 08100 Nuoro, Italy; anna.calvisi@aslnuoro.it; 9 SOC Ematologia Azienda USL-IRCSS di Reggio Emilia, 42122 Reggio Emilia, Italy; isabella.capodanno@ausl.re.it; 10 Cancer Center, IRCCS Humanitas Research Hospital, Humanitas University, 20089 Rozzano, Italy; matteo.della_porta@hunimed.eu; 11 Unit of Hematology and Bone Marrow Transplantation, IRCCS San Raffaele Scientific Institute, 20132 Milan, Italy; diral.elisa@hsr.it; 12 Department of Oncology and Onco-Hematology, University of Milan, 20122 Milan, Italy; bruno.fattizzo@policlinico.mi.it; 13 Haematology Department, San Giovanni-Addolorata Hospital, 00184 Rome, Italy; sfenu@hsangiovanni.roma.it; 14 Istituto di Ematologia Seràgnoli, IRCCS Azienda Ospedaliero, Universitaria di Bologna, 40138 Bologna, Italy; stefania.paolini@unibo.it (S.P.); carlo.finelli@unibo.it (C.F.); 15 Department of Clinical and Experimental Medicine, University of Sassari, 07100 Sassari, Italy; cfozza@uniss.it; 16 Hematology, Hospital Città della Salute e della Scienza, 10126 Turin, Italy; cfrairia@cittadellasalute.to.it (C.F.); vgiai@cittadellasalute.to.it (V.G.); 17 Division of Hematology, Ospedale Valduce, 22100 Como, Italy; mturrini@valduce.it; 18 Istituto di Ematologia, Università di Sassari, 07100 Sassari, Italy; mariantonia.isoni@aousassari.it; 19 Division of Internal Medicine and Hematology, San Luigi Gonzaga Hospital, 10043 Orbassano, Italy; f.itri@sanluigi.piemonte.it; 20 Hematology Division, UOSD Malattie Mieloproliferative, University of Rome Tor Vergata, 00133 Rome, Italy; luca.maurillo@uniroma2.it; 21 Hematology, ASST Cremona, 26100 Cremona, Italy; alfredo.molteni@asst-cremona.it; 22 Unità Operativa di Ematologia con TMO AOU “Policlinico—San Marco”, Dipartimento di Scienze Mediche, Chirurgiche e Tecnologie Avanzate “G.F. Ingrassia”, University of Catania, 95123 Catania, Italy; palumbo.ga@gmail.com; 23 Department of Hematology, Spedali Civili di Brescia, 25123 Brescia, Italy; annamaria.pelizzari@asst-spedalicivili.it; 24 Hematology and Transplant Center, Ospedale Oncologico di Riferimento Regionale “Armando Businco”, Azienda Ospedaliera Brotzu, 09121 Cagliari, Italy; federica.pilo@aob.it; 25 Hematology, Università Politecnica Marche, AOU Marche, 601221 Ancona, Italy; a.poloni@staff.univpm.it; 26 Ematologia e Centro Trapianti, Ospedale Guglielmo da Saliceto, 29121 Piacenza, Italy; c.bosi@ausl.pc.it; 27 UOC Ematologia Fondazione Casa Sollievo della Sofferenza, 71013 San Giovanni Rotondo, Italy; g.sanpaolo@operapadrepio.it; 28 Hematology/Bone Marrow Transplantation Unit, Ospedale dell’Angelo and Ospedale SS. Giovanni e Paolo, 30174 Venezia, Italy; rosaria.sancetta@aulss3.veneto.it; 29 MDS Unit, Hematology, DMSC, AOU Careggi, University of Florence, 50134 Florence, Italy; cristinaamato@virgilio.it (C.A.); valeria.santini@unifi.it (V.S.); 30 Department of Biomedicine and Prevention, University of Rome Tor Vergata, 00133 Rome, Italy; voso@med.uniroma2.it; 31 School of Life and Medical Sciences, University of Hertfordshire, Hatfield AL10 9AB, UK; sssalek52@gmail.com; 32 St. Petersburg State University Hospital, St. Petersburg 190103, Russia; tation16@gmail.com; 33 Hematology Department, London North West University Healthcare NHS Trust, London HA1 3UJ, UK

**Keywords:** myelodysplastic neoplasms, quality of life, transfusion dependency, caregivers, unmet needs

## Abstract

Background/Objectives: The aim of this study was to assess the unmet needs of myelodysplastic neoplasm (MDS) patients and their caregivers, focusing on how these needs impact quality of life (QoL) and daily functioning. MDS predominantly affects older adults. It is often complicated by severe red blood cell transfusion-dependent anemia and may require frequent hospital visits, conferring a substantial burden on patients and caregivers. Methods: A national survey was conducted between June 2022 and May 2023 in 46 hematology centers across Italy, involving 259 patients and 105 caregivers. The survey included validated QoL tools (QOL-E and HM-PRO) to measure the impact of disease and treatments on health-related QoL and symptoms. Results: Of the 259 patients surveyed, 42% were transfusion-dependent, with 45% reporting distress related to hospital travel, which was significantly associated with lower QoL scores (QOL-E physical score 50.0 vs. 62.5, *p* < 0.001). Transfusion dependency led to worse outcomes across physical, emotional, and social domains (HM-PRO Part A score 59.8 vs. 23.7, *p* < 0.001). Anxiety affected 66% of patients, while 56% reported feeling emotionally distressed. Forty-eight percent of patients required a caregiver, and among caregivers, 29% reported significant disruption to their work, including changing their job or reduced hours. Patients requiring frequent hospital visits showed notably worse QoL scores (HM-PRO emotional score 56.8 vs. 31.8, *p* < 0.001). Conclusions: This study identified substantial unmet needs for MDS patients, particularly in addressing the heavy burden of transfusions and hospital visits. Both patients and caregivers experienced significant impact on daily life and on QoL, highlighting the urgent need for treatments that reduce hospital dependency, improve patient outcomes, and alleviate the caregiver burden.

## Simple Summary

In the present study, we examined the unmet needs of myelodysplastic neoplasm (MDS) patients and their caregivers, focusing on the impact on their quality of life (QoL). MDS affects mainly older adults, and it is often complicated by severe red blood cell transfusion-dependent anemia and may require frequent hospital visits, disrupting daily life. In this national survey undertaken in Italy, data were collected from 259 patients and 105 caregivers. Forty-two percent of patients were transfusion-dependent, and 45% found hospital travel physically and economically burdensome. In addition, 66% of patients reported anxiety, and 56% felt distressed about their condition. Caregivers (36% of whom were involved in patient care) reported a significant impact on their work and personal lives. These findings highlight the need for improved treatments for transfusion-dependent anemia that reduce transfusion frequency and hospital visits. Our results may help guide healthcare professionals to better meet the needs of MDS patients and their caregivers, ultimately improving their QoL.

## 1. Introduction

Health-related quality of life (HRQoL) is a complex, multidomain variable construct that represents the patient’s overall perception of the impact of an illness and its treatment [1,2]. Myelodysplastic neoplasms (MDS) are clonal diseases characterized by ineffective hematopoiesis, leading to peripheral blood cytopenias and an increased risk of progression to acute myeloid leukemia (AML) [3].

MDS predominates in the elderly with a median age of diagnosis ≥ 70 years old [4]. In clinical practice, a cutoff of 3.5 for the Revised International Prognostic Scoring System (IPSS-R) score defines 2 distinct risk groups: lower-risk MDS (score ≤ 3.5, median survival 5.9 years) and higher-risk MDS (score > 3.5, median survival 1.5 years) [5]. This risk stratification has been the basis of a patient-centered approach to the management of MDS that aims to improve cytopenias for patients with lower-risk disease and to prevent or delay progression to AML for higher-risk patients. Currently, several treatment options are available for patients with MDS: hypomethylating agents (e.g., azacytidine), hematopoietic stimulating agents (e.g., erythropoietin, G-CSF, and luspatercept), supportive care (e.g., blood and platelet transfusions and antibiotics), immunomodulatory agents (e.g., lenalidomide and cyclosporine), low-dose or intensive chemotherapy, and iron overload chelation (deferasirox) [6,7]. However, at present, the only curative treatment for MDS is allogeneic hematopoietic stem cell transplantation (alloHSCT), an option for a limited number of younger and fit MDS patients either at higher risk or with significant cytopenias [8].

Therefore, maintaining/improving HRQoL is one of the main goals of treatment both for lower- and higher-risk MDS patients [9].

There are many factors related to MDS and unmet needs that may impact the QoL of patients and their caregivers. Since patients with MDS are typically elderly, they often present with one or more comorbidities at the time of diagnosis, which can contribute significantly to their HRQoL [10,11]. However, the proportion of problems in usual activities, such as limitations in mobility, self-care, pain/discomfort, and anxiety/depression, is significantly higher in MDS patients in comparison to age- and sex-matched peers [12].

Several factors contribute to this, including the need for blood transfusions and frequent hospital visits in patients with anemia, as well as complications like infections and bleeding due to neutropenia and thrombocytopenia [13,14]. Among the most prominent symptoms of MDS is fatigue, which is noteworthy as it can only be assessed through patient self-reporting [15]. Fatigue includes a range of symptoms, such as physical weakness, lethargy, and diminished mental alertness. These issues can profoundly affect both life expectancy and quality of life (QoL) [5,12,16]. However, fatigue is not entirely correlated with hemoglobin levels [17]. Indeed, the multifaceted impact of MDS on HRQoL extends well beyond fatigue, including a range of physical, emotional, and functional challenges [9,18,19]. Although chronic fatigue remains the most reported and debilitating symptom, patients also experience pain, restricted mobility, dyspnea, mood disorders, sleep disturbances, and nutritional imbalances, all of which contribute to increased frailty and reduced autonomy [12,20]. These issues are often exacerbated by treatment side effects and comorbidities.

Beyond physical symptoms, HRQoL in MDS patients is also shaped by psychosocial and emotional factors, including limited communication with physicians, lack of understanding about the disease, fear of progression to acute leukemia, and anxiety with regard to mortality. Comprehensive assessment tools such as the QUALMS, HM-PRO, and QOL-E have been validated to evaluate HRQoL in MDS patients, and the importance of the integration of these tools in routine care is recognized [9].

In addition, MDS patients often need a caregiver to support them, whose life can be affected by this role [21,22].

We performed a national Italian survey to explore the impact of MDS on patients and caregivers across different aspects of everyday life. Moreover, we aimed to explore patient-reported outcomes (PROs) through validated measures. Finally, this survey collected patients’ preferences and unmet needs in Italy.

## 2. Materials and Methods

### 2.1. Study Design

The survey was promoted by the Associazione Italiana Pazienti Sindromi Mielodisplastica (AIPaSiM) advocacy group from June 2022 to May 2024 in 46 different hematology centers in Italy. The survey was available online and also distributed in paper version to patients attending the centers. It included items that were identified by hematologists and patients: 60 items relevant to patients and 20 relevant to caregivers. The PRO measures (PROMs), QOL-E, and HM-PRO were included in the survey.

### 2.2. QOL-E

The QOL-E is the first HRQoL instrument developed and validated specifically for MDS patients [23]. It consists of 29 items, two of which do not fit into a multi-item scale and assess subjects’ general perception of well-being at the time of the assessment and compare it with that from a month earlier (i.e., Item 1 and Item 2, respectively). The remaining 27 items address the following six domains:

- Physical well-being (QOL-FIS): four items (Items 3a–d).
- Functional well-being (QOL-FUN): three items (Items 4a–b and Item 5).
- Social and family life (QOL-SOC): four items (Items 6a–c and Item 7).
- Sexual well-being (QOL-SEX): two items (Item 8 and Item 14f).
- Fatigue (QOL-FAT): seven items (Item 9, Item 10, Items 11a–d, and Item 12).
- MDS-specific disturbances (QOL-SPEC): seven items (Item 13, Items 14a–e, and Item 14g).

Three summary scores were also derived from these six domains:

- General (QOL-GEN): the average of all domains, except for QOL-SPEC.
- General (QOL-GENV): the average of all domains, except for QOL-SPEC and QOL-SEX.
- QOL-ALL: the average of QOL-GEN and QOL-SPEC.
- QOL-ALLV: the average of QOL-GEN and QOL-SPEC, except for QOL-SEX
- Treatment Outcome Index (QOL-TOI): the average of QOL-FIS, QOL-FUN, and QOL-SPEC.

Each domain or summary score of the QOL-E questionnaire is scored using a standardized scale with a possible range of 0–100. A higher score indicates better health or lower symptomology for that domain or summary score (i.e., lower scores reflect worse outcomes).

### 2.3. HM-PRO

The HM-PRO (Hematological Malignancy-Patient-Reported Outcomes) is a validated patient-reported outcome tool designed specifically to assess the impact of hematological malignancies, such as MDS, leukemia, lymphoma, and myeloma, on patients’ HRQoL and symptoms [24,25,26,27]. It consists of the following two parts: assessing impact (Part A) and signs and symptoms (Part B) of hematological malignancies. Part A has a total of 24 items in four domains as follows: physical behavior, social behavior, emotional behavior, and eating and drinking habits. Patients’ responses are recorded on a three-point Likert scale (not at all, a little, and a lot) and “not applicable” as a separate response option.

Part B consists of 18 items in a single domain, and the responses are captured on a three-point severity Likert scale (not at all, mild, and severe). The scales have linear scoring systems ranging from 0 to 100, with higher scores representing greater (negative) impact (worse outcomes) on QoL and symptom burden.

### 2.4. Statistical Analysis

The median value and interquartile range (IQR) were used for reporting continuous variables, while the absolute and relative frequency were reported for categorical variables.

Comparisons between groups were performed using Fisher’s exact test, the Mantel-Haenszel chi-square test, or Wilcoxon’s two-sample test as appropriate, and Student’s two-sample *t*-test for normal continuous variables.

The consistency of the QOL-E items was evaluated through Cronbach’s alpha coefficient. A *p*-value of ≤0.05 was considered statistically significant. All statistical analyses were carried out using SPSS Software for Windows (version 13, SPSS Inc., Chicago, IL, USA).

## 3. Results

From June 2022 to May 2023, 259 patients and 105 caregivers completed the survey.

### 3.1. Patient Characteristics

A summary of the main patient characteristics is presented in Table 1. The median age of the patients was 73 years (IQR: 64–79), and 56% were female. MDS disease duration was less than 1 year for 45 patients (18%), 1–2 years for 59 patients (24%), 2–5 years for 73 patients (30%), and over 5 years for 69 patients (28%).

Data on treatment was available for 244 patients. One hundred and sixty-seven patients (70%) were receiving the following treatment: 100 (41%) with recombinant erythropoietin, luspatercept, lenalidomide, danazol, or eltrombopag for lower-risk MDS and 53 (22%) with azacytidine or chemotherapy for higher-risk MDS. Five patients (2%) received an allogeneic bone marrow transplantation, and 11 patients (5%) did not know what the ongoing treatment was. Overall, 97 patients (42%) received regular red blood cell transfusions.

MDS affected work life in 20% (N = 53) of patients, leading to changes in their job type (N = 3) or working hours (N = 25) or to retirement/activity or resignation (N = 25). At the time of the survey, only 60 patients (23%) were still employed.

It is worth noting that almost half of all patients (N = 118; 48%) needed a caregiver to accompany them to the hospital. Fifty-five patients (21.5%) lived in a different area (N = 41) or in a different region (N = 14) from the treating hematological center. Overall, for 107 patients (45%), traveling to the hospital was claimed as being distressful in terms of physical and economic burden and impacted family members and their employment.

### 3.2. PROM QOL-E and HM-PRO

A total of 211 (84.7%) patients responded to QOL-E and HM-PRO questionnaires, although some patients only answered part of the questions. Median scores across all domains are presented in Table 2.

MDS affected different domains of everyday life of patients, particularly fatigue (median score of 76.2) and sexual and functional domains (median scores of 66.6 for both), as presented in Table 2. For instance, 47% of patients found it difficult to work or study and the same proportion to go on holiday, 56% felt distressed and 66% anxious, and 60% worried about their treatment.

### 3.3. Impact of Distance from Hospital and Time Spent in Hospital on HRQoL

A total of 114 patients (63%) reported that their dependence on hospitals, physicians, or nurses disturbed their everyday life.

For 107 patients (45%), traveling to the hospital was claimed as being distressful. In this group, there were more patients living distant from the hospital (32% vs. 13%, *p* = 0.002), needing a family member, a volunteer, or a friend to go to the treating center (63% vs. 36%, *p* < 0.001), and receiving transfusions (53.5% vs. 35%, *p* = 0.006) compared to the remaining 55% of patients.

Comparing PROMs of these patients to the others, distress was associated with significantly worse median HM-PRO physical (PB, 53.5 vs. 21.4, *p* < 0.001), emotional (EB, 56.8 vs. 31.8, *p* < 0.001), social (SB, 66 vs. 50, *p* < 0.001), eating and drinking habits (ED, 25 vs. 25, *p* < 0.001), Part A (56.0 vs. 26.6, *p* < 0.001) and Part B symptom score (SS, 20.6 vs. 8.8, *p* < 0.001) and QOL-E physical (Ph, 50.0 vs. 62.5, *p* < 0.001), functional (Fun, 22.2 vs. 88.8, *p* < 0.001), social (Soc, 25.0 vs. 75.0, *p* < 0.001), fatigue (Fat, 64.3 vs. 80.9, *p* < 0.001), general (Gen, 46.7 vs. 72.1, *p* = 0.003), MDS specific (MDSS, 44.04 vs. 73.8, *p* < 0.001), and treatment-outcome index scores (TOI, 38.2 vs. 69.8, *p* < 0.001) (Table 3).

**Figure 1:**
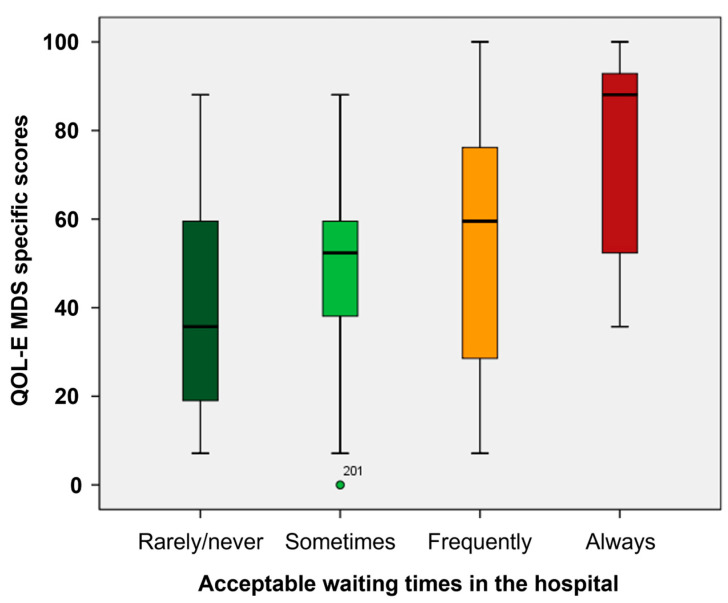
Box plot of QOL-E MDS-specific score according to waiting times in hospital. The central line denotes the median value (50th percentile), while the box contains the 25th to 75th percentiles of the dataset. The whiskers mark the 5th and 95th percentiles, and values beyond these upper and lower bounds are considered outliers, marked with dots. Outlier (green dot) is shown.

Moreover, waiting times in the hospital were regarded as unacceptable for 87 patients (37%), who in fact had worse median scores in HM-PRO EB (*p* < 0.001), Part A (*p* = 0.020), SS scores (*p* = 0.002), QOL-E Fun (*p* = 0.004), Fat (*p* < 0.001), Ses (*p* = 0.018), MDSS (*p* = 0.001), Gen (*p* = 0.030), and TOI (*p* = 0.003) (Figure 1 and Supplementary Table S1).

Telemedicine communication was preferred in 54% of cases, particularly if reaching the hospital was reported as being distressful (62% yes vs. 45% no, *p* = 0.035).

### 3.4. Impact of Treatment and Transfusion on HRQoL

PROM scores were not significantly different across the different treatment groups. Only 18% of patients felt that treatment negatively impacted their everyday life, and this aspect was independent of the type of treatment but was associated with red blood cell transfusion dependency. Overall, receiving regular transfusions negatively impacted HRQoL (Table 4), and 47% felt that transfusion dependence affected their everyday life.

Indeed, for 70% (49/70) of transfusion-dependent patients, treatment impacted everyday life negatively compared to 49% (46/94) of transfusion-independent patients (*p* = 0.011) and had significantly worse scores across all domains of QOL-E and HM-Pro (Supplementary Table S2).

**Figure 2:**
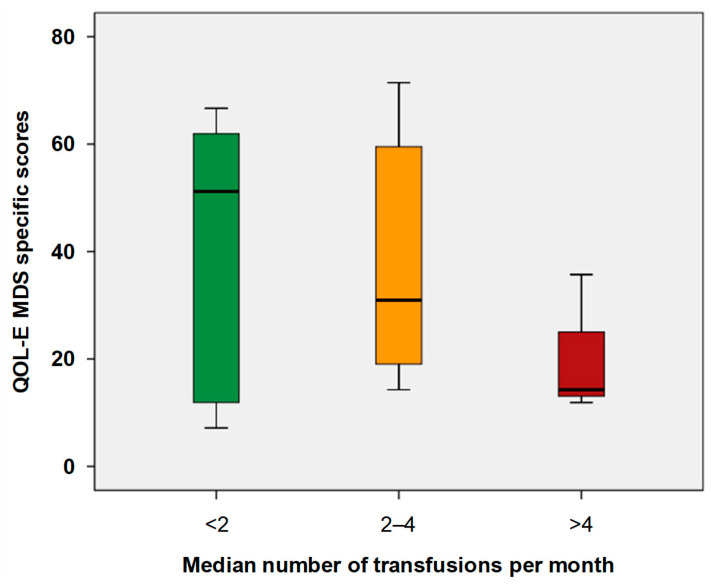
Box plot of QOL-E MDS-specific scores according to transfusion frequency. The central line denotes the median value (50th percentile), while the box contains the 25th to 75th percentiles of the dataset. The whiskers mark the 5th and 95th percentiles.

The increasing frequency of transfusions was associated with worse HM-PB (*p* = 0.037), SB (*p* = 0.038), Part A scores (*p* = 0.029), QOL-E Soc (*p* = 0.012), Fat (*p*= 0.034), MDSS (*p* = 0.002), and Gen scores (*p* = 0.049) (Figure 2).

Only 2 patients received transfusions at home, but 38% (N = 30) would prefer receiving transfusions at home. Moreover, 77% of those with distress reaching the hospital and 20% of those far from the treating center had worse PROM scores (Table 4). QOL-E and HM-PRO scores for the patients’ preferred place to undertake transfusions are summarized in Table 5.

Thirty-five patients (17%) felt that they were a burden for their family and had lower QOL-E and higher HM scores across all domains (*p* < 0.001; Supplementary Table S3). In this subgroup, there was a higher proportion of transfusion-dependent patients (61% vs. 35%, *p* = 0.026), a greater frequency of patients for whom MDS impacted their work life (36% vs. 21%, *p* = 0.013), and more patients needing a caregiver (39% vs. 17%, *p* = 0.006).

### 3.5. Work Life

Retired patients had worse PROM median scores as follows: QOL-E Fis (*p* = 0.014), Soc (*p* = 0.030), Gen (*p* = 0.043), all (*p* = 0.040), and TOI (*p* = 0.012), HM-PRO PB (*p* = 0.005), SW (*p* < 0.001), and PART A (*p* = 0.001).

Ninety-seven percent of patients, whose work–life balance was affected by MDS, reported that fatigue interfered with their daily activity, as reflected by QOL E scores.

### 3.6. Patients’ Preferences and Communication with Physician

For 18% of patients (N = 43), the communication of diagnosis was incomplete, and for 38% (N = 92), communication was partially understood or misunderstood. Twenty-four percent of patients (N = 50) were not satisfied with communication of their treatment program and expected side effects and subsequently searched for information on websites (34%) or from other doctors (30%). In contrast, 85% of patients were satisfied with the nurse’s support.

The worst aspect of their treatment journey was waste of time for 37% of patients (N = 86) and hospital disorganization for 20% (N = 46).

The most important unmet needs reported by patients for escalation to the authorities were access to new medicines for 35%, faster access to treatments for 33%, and being treated in their own area for 12%. Other unmet needs were comprehension of diagnosis for 15% and treatment options for 21%.

### 3.7. Caregivers

Thirty-six percent of the patients who responded to the survey had a caregiver.

One hundred and five caregivers, of whom 94% were family members (35% partners and 59% other family components) and 80% were females, completed the survey. The median age of caregivers was 56 years (IQ range: 48–64), and 53% were still employed.

The caregiver’s work life was largely affected by their role, leading to a change in type of job (29% of cases), or working hours (30%), or to work discontinuation (14%). On a scale of 0–10 on the impact on work–life balance, family caregivers scored a median of 4 (interquartile range: 2–6).

## 4. Discussion

This national survey of MDS patients and their caregivers offers valuable insights into the disease burden and its profound impact on HRQoL. Using two validated questionnaires, the QOL-E and HM-PRO, this study highlights several key areas that affect HRQoL in MDS patients, including fatigue, transfusion dependency, and emotional well-being, as well as the significant impact on caregivers’ QoL. These findings corroborate results from recent studies that highlight the complex relationship between physical symptoms, emotional distress, and the practical burdens associated with chronic disease management.

Patients with MDS in this study were elderly (median of 73 years), 42% were transfusion dependent and therefore also dependent (in 48% of cases) on caregivers to accompany them to the hospital.

Fatigue was identified as a major factor in reducing HRQoL, with over 76% of patients in this study reporting it as a significant problem. This finding is consistent with other studies [22,28,29], including a study by Efficace et al. that investigated factors associated with fatigue severity in 280 newly diagnosed higher-risk MDS patients [17]. In that study, fatigue severity strongly impacted overall QoL, particularly in women. Another study by Escalante and colleagues based in the United States evaluated fatigue, symptom burden, and health-related QoL in 303 patients, including 145 with MDS [29]. In the MDS group of patients, severe fatigue was reported, with a mean fatigue score (FACT-F questionnaire) of 25 (i.e., severe). The average QoL score (FACT-G questionnaire) was 69. Common strategies for managing fatigue included conserving energy, physical activity, and naps. Further research is needed to assess the effectiveness of these strategies in improving outcomes for MDS patients.

Another key finding that emerged from our study was the significant degree of emotional distress experienced by MDS patients, with 66% reporting anxiety related to their disease and treatment. This is consistent with the recent literature that emphasizes the psychosocial impact of MDS [12]. In addition, a large-scale study by Stauder and colleagues revealed that patients experience significant impairment in HRQoL, particularly in pain/discomfort, mobility, anxiety/depression, and usual activities [12]. These limitations were more pronounced in older individuals, females, and those with high comorbidity, low hemoglobin, or transfusion needs, compared to age- and sex-matched norms.

Transfusion dependency is considered a major factor that negatively impacts HRQoL. In this study, 42% of patients were dependent on regular red blood cell transfusions, and these patients reported significantly worse outcomes across multiple HRQoL domains. This finding is supported by other studies showing the impact of transfusion dependency on HRQoL [30]. Fenaux et al. demonstrated that transfusion dependency is associated with increased fatigue, reduced physical function, and greater emotional distress [31]. Novel therapies such as luspatercept and imetelstat, which have been shown to reduce transfusion needs, have been shown to improve QoL [32].

Our findings also highlighted the importance placed on caregivers, many of whom experienced significant disruptions to their work and personal lives. Almost half (48%) of patients required assistance from caregivers for hospital visits, and caregivers reported high levels of stress and emotional strain. This is consistent with recent studies that have examined the impact of caregiving in hematologic malignancies, including MDS [22,33,34].

A large real-world study combined surveys with patient chart reviews from 1445 patients to document real-world clinical practice and the burden of MDS clinical practice [22]. It found discrepancies between patient and physician reports on symptoms and QoL, with patients reporting higher fatigue and symptom frequency. Caregiver burden was also underestimated by physicians. The findings highlight the need for improved therapeutic strategies and better communication between patients, caregivers, and healthcare providers to enhance MDS management and QoL. Addressing caregiver needs, including providing support for managing the logistical and emotional challenges of caregiving, is essential to improving the overall care experience for MDS patients.

One of the most striking findings of our study was the significant distress caused by hospital visits and waiting times, with 37% of patients reporting dissatisfaction with the amount of time spent in the hospital. These logistical challenges were associated with worse HRQoL outcomes, particularly in patients who had to travel long distances or rely on caregivers for transportation. This finding is in line with several studies that the physical and economic burden of frequent hospital visits can significantly contribute to patient and caregiver distress [35,36,37]. Telemedicine and home-based care options could help alleviate these burdens, particularly for patients who live far from treatment centers.

A recent study by LoCastro and colleagues assessed the feasibility and usability of a telehealth-delivered Serious Illness Care Program for patients with AML and MDS [38]. With a 95% retention rate and usability scores averaging 5.9/7, the program was well-received. Most participants found it valuable, reporting enhanced closeness with clinicians and alignment in curability and life expectancy estimates. Furthermore, 89.5% of patients would recommend the telehealth program, demonstrating its acceptability and promise for wider use.

In terms of treatment burden, only 18% of patients felt that their current treatment had a negative impact on their everyday life. This finding is somewhat surprising, given the high level of physical and emotional distress reported by patients regarding the impact of transfusion. However, it may reflect the fact that many patients are on supportive therapies, such as erythropoiesis-stimulating agents or low-intensity chemotherapy, which are generally well-tolerated. Nonetheless, the negative impact of transfusion dependency on HRQoL suggests that efforts to reduce the need for transfusions, either through more effective anemia management or through the development of novel therapies, should remain a key focus in MDS treatment [13,39,40].

## 5. Study Limitations

While this study provides valuable insights into the impact that MDS has on patients and their caregivers, several limitations need to be highlighted. First, the survey design may introduce response bias, as patients who are more engaged in their care may be more likely to participate. Second, the use of PROs inherently involves subjective assessments, which may vary between individuals and may not fully capture the complexity of disease burden. In addition, the cross-sectional nature of this study limits the ability to assess changes in HRQoL over time. Future longitudinal studies will be essential for tracking how HRQoL evolves with disease progression and treatment.

## 6. Conclusions

This Italian study emphasizes the significant challenges faced by patients with MDS and their caregivers. The emotional and economic burden of frequent hospital visits, long waiting times, and transfusion dependency negatively impacts QoL for both groups. Fatigue, distress, and the high frequency of transfusions are major factors contributing to worse QoL for patients. This study highlights the importance of improving communication from physicians with reinforcement from nurse case managers and advanced practice nurses regarding diagnosis, prognosis, and treatment options, while also identifying unmet needs that influence patient care. To address these issues, there is a need for interventions that reduce transfusion requirements, enable treatment at home, and improve access to novel therapies. Telemedicine and strategies to alleviate the logistical burden of hospital visits could further enhance patients’ HRQoL and support caregivers. 

## Figures and Tables

**Table 1:** Patient characteristics.

Patient Characteristics (N = 259)	Median (IQR)	N (%)
Age	73.0 (64–79)	
Sex		
M		114 (44.0)
F		145 (56.0)
Time from diagnosis		
<1 year		45 (18.3)
1–2 years		59 (24.0)
2–5 years		73 (29.7)
5–10 years		46 (18.7)
>10 years		23 (9.3)
Missing		13
Ongoing treatment		
Erythropoietin		68 (27.9)
Azacytidine		40 (16.4)
Lenalidomide		14 (5.7)
Hydroxycarbamide		6 (2.5)
Luspatercept		16 (6.6)
Chemotherapy		9 (3.7)
HSCT		5 (2.0)
Eltrombopag		1 (0.4)
Danazol		1 (0.4)
Unknown		11 (4.5)
No treatment		73 (29.9)
Transfusion dependence		
Yes		97 (42.4)
No		132 (57.6)
Missing		30
Median number of RBC unit/month		
<2		33 (33.0)
2–4		54 (54.0)
>4		13 (13.0)

HSCT, hematopoietic stem cell transplantation; IQR, interquartile range; RBC, red blood cells.

**Table 2:** QOL-E and HM-PRO scores of the MDS patients who completed PROM surveys.

QOL-E Index		HM-PRO Scores	
Domain (N)	Median Score (IQR)	Domain (N)	Median Score (IQR)
Physical (188)	62.5 (37.5–75.0)	Physical (211)	35.7 (7.1–65.5)
Function (158)	66.6 (22.2–100)	Emotional (209)	40.9 (13.6–59.1)
Social (158)	50.0 (25.0–100)	Social (211)	50.0 (0–66.7)
Sexual (90)	66.6 (41.6–100)	Eating and drinking habits (209)	25.0 (0–50.0)
Fatigue (183)	76.2 (54.5–90.5)	Part A (211)	34.3 (13.7–59.7)
MDS-specific (131)	59.5 (34.5–88.1)	Part B (196)	17.6 (2.9–26.4)
General (62)	58.2 (43.8–86.3)		
Treatment outcome index (101)	62.9 (34.5–83.5)		
All (54)	62.5 (36.7–86.1)		

HM-PRO, hematological malignancy-patient-reported outcomes; IQR, interquartile range; QOL-E, psychometric questionnaire assessing HRQoL in MDS patients; MDS, myelodysplastic neoplasms.

**Table 3:** QOL-E and HM-PRO scores according to reported distress traveling to the hospital.

		Percentiles			
		25	Median	75	*p*-Value
PB_SCORE	No	0.0	21.4	50.0	<0.001
	Yes	35.7	53.6	71.4	
EB_SCORE	No	9.1	31.8	45.5	<0.001
	Yes	30.7	56.8	67.0	
SW_SCORE	No	0.0	50.0	66.7	<0.001
	Yes	33.3	66.7	83.3	
ED_SCORE	No	0.0	25.0	25.0	<0.001
	Yes	0.0	25.0	50.0	
PARTA_SCORE	No	6.3	26.6	52.3	<0.001
	Yes	31.8	56.0	68.8	
SS_SCORE	No	0.0	8.8	20.6	<0.001
	Yes	12.5	20.6	31.6	
QOL-FIS	No	50.0	62.5	87.5	<0.001
	Yes	37.5	50.0	71.9	
QOL-FUN	No	22.2	88.9	100.0	<0.001
	Yes	22.2	22.2	63.9	
QOL-SOC	No	37.5	75.0	100.0	<0.001
	Yes	15.6	25.0	71.9	
QOL-FAT	No	66.7	81.0	90.5	<0.001
	Yes	57.1	64.3	76.2	
QOL-SPEC	No	50.0	73.8	92.9	<0.001
	Yes	19.0	44.0	61.3	
QOL-GEN	No	53.8	72.0	88.1	0.003
	Yes	32.3	46.7	65.8	
QOL-GENV	No	53.5	73.4	91.4	<0.001
	Yes	34.8	40.1	66.5	
QOL-ALL	No	53.2	71.3	88.6	0.005
	Yes	33.0	43.3	64.4	
QOL-ALLV	No	52.4	77.3	90.5	<0.001
	Yes	33.0	42.2	64.7	
QOL-TOI	No	55.2	69.8	87.7	<0.001
	Yes	30.4	38.3	65.2	

HM-PRO, hematological malignancy-patient-reported outcomes; QOL-E, a psychometric questionnaire assessing HRQoL in MDS patients. QoL, quality of life; QOL-ALL, calculated by taking the mean of QOL-GEN and QOL-MDSS; QOL-ALLV, calculated by taking the mean of QOL-GEN and QOL-SPEC without QOL-SEX domain included; QOL-FAT, fatigue; QOL-FIS, physical well-being; QOL-FUN, functional well-being; QOL-GEN, calculated by taking the mean of all domains except for QOL-SPEC; QOL-GENV, calculated by taking the mean of all domains except for QOL-SPEC and QOL-SEX domain included; QOL-SEX, sexual well-being; QOL-SOC, social/family well-being; QOL-SPEC, MDS-specific disturbances; QOL-TOI, treatment outcome index calculated by taking the mean of QOL-FIS, QOL-FUN, and QOL-SPEC.

**Table 4:** QOL-E and HM-PRO scores according to transfusion dependence.

		Percentiles			
		25	Median	75	*p*-Value
PB_SCORE	Yes	32.1	57.1	78.6	<0.001
	No	0.0	21.4	37.9	
EB_SCORE	Yes	45.5	56.8	69.3	<0.001
	No	9.1	22.7	43.2	
SW_SCORE	Yes	33.3	66.7	100.0	<0.001
	No	0.0	0.0	66.7	
ED_SCORE	Yes	18.8	50.0	62.5	<0.001
	No	0.0	0.0	25.0	
PARTA_SCORE	Yes	35.3	59.8	70.7	<0.001
	No	4.3	23.7	39.8	
SS_SCORE	Yes	14.0	19.1	30.1	0.044
	No	0.0	11.8	20.6	
QOL-FIS	Yes	37.5	50.0	62.5	<0.001
	No	43.8	62.5	93.8	
QOL-SOC	Yes	25.0	25.0	50.0	<0.001
	No	43.8	87.5	100.0	
QOL-FAT	Yes	56.0	64.3	77.4	<0.001
	No	66.7	81.0	90.5	
QOL-SPEC	Yes	14.3	31.0	59.5	<0.001
	No	54.8	81.0	92.9	
QOL-GEN	Yes	31.9	41.1	65.5	<0.001
	No	49.0	73.4	91.5	
QOL-ALL	Yes	30.0	40.1	64.2	<0.001
	No	50.1	77.3	90.6	
QOL-TOI	Yes	27.9	35.1	70.4	<0.001
	No	44.2	68.5	92.5	

HM-PRO, hematological malignancy-patient-reported outcomes; QOL-E, psychometric questionnaire assessing HRQoL in MDS patients. QOL, quality of life; QOL-ALL, calculated by taking the mean of QOL-GEN and QOL-MDSS (MDS-specific disturbances); QOL-FAT, fatigue; QOL-SPEC, MDS-specific disturbances; QOL-FIS, physical well-being; QOL-GEN, calculated by taking the mean of all domains except for QOL-SPEC; QOL-GENV, calculated by taking the mean of all domains except for QOL-SPEC and QOL-SEX domain included; QOL-SEX, sexual well-being; QOL-SOC, social/family well-being; QOL-SPEC, MDS-specific disturbances; QOL-TOI, treatment outcome index calculated by taking the mean of QOL-FIS, QOL-FUN, functional well-being, and QOL-SPEC.

**Table 5:** QOL-E and HM-PRO scores according to place preferred for transfusions.

	Preferred Place for Transfusion		
	Home	Hospital	*p*-Value
HM-PRO scores; median (IQR)			
Emotional	59 (29–68)	50 (25–50)	0.045
Part B	31 (19–52)	16 (7–27)	0.008
QOL-E scores; median (IQR)			
Functional	25 (11–44)	37 (17–47)	0.006
Fatigue	57 (26–39)	64 (30–42)	0.007
MDS specific	27 (10–30)	32 (19–35)	0.012

IQR, interquartile range; MDS, myelodysplastic neoplasms.

## Data Availability

The original contributions presented in this study are included in the article/Supplementary Material. Further inquiries can be directed to the corresponding author.

## Supplementary Materials

The following supporting information can be downloaded at: https://www.mdpi.com/article/10.3390/cancers17091587/s1, Supplementary Table S1. QOL-E and HM-PRO scores according to acceptable waiting time in hospital; Supplementary Table S2. QOL-E and HM-PRO scores according to negative impact of treatment on everyday life; Supplementary Table S3. QOL-E and HM-PRO scores according to patients feeling to be a burden for their family.

## Abbreviations

The following abbreviations are used in this manuscript:

AIPaSiM	Associazione italiana pazienti sindromi mielodisplastica
alloHSCT	allogeneic hematopoietic stem cell transplantation
HM-PRO	hematological malignancy-patient-reported outcomes
HRQoL	health-related quality of life
HSTC	hematopoietic stem cell transplantation
IPSS-R	Revised International Prognostic Scoring System
IQR	interquartile range
MDS	myelodysplastic neoplasms
MDSS	MDS-specific
PRO	patient-reported outcome
PROM	PRO measure
QOL-E	HRQoL instrument developed specifically for MDS patients
