# Supplementary material for: Unmet Needs and Their Impact on Quality of Life and Symptoms in Myelodysplastic Neoplasm Patients and Caregivers
Source: Cancers (Basel). 2025 May 7;17(9):1587. doi: 10.3390/cancers17091587 (PMC12072108; doi:10.3390/cancers17091587)
Supplement: Supplementary file 1 [file cancers-17-01587-s001.zip › cancers-3583974-supplementary.pdf]

Supplementary Table S1. QOL-E and HM-PRO scores according to acceptable waiting time in hospital

Was waiting time in hospital acceptable?		Percentiles 25	Median	75	p value
PB_SCORE	Never	7.1	60.7	92.9	0.279
	Rarely	7.1	71.4	82.1	
	Sometimes	32.9	42.9	53.6	
	Almost always	19.6	41.7	64.3	
	Always	7.1	57.1	71.4	
EB_SCORE	Never	33.0	68.2	89.8	0.001
	Rarely	47.7	59.1	62.5	
	Sometimes	11.4	54.5	65.9	
	Almost always	17.0	38.2	59.1	
	Always	13.6	27.3	45.5	
SW_SCORE	Never	20.8	108.3	158.3	0.053
	Rarely	25.0	6.7	100.0	
	Sometimes	41.7	6.7	6.7	
	Almost always	0.0	50.0	6.7	
	Always	0.0	6.7	100.0	
ED_SCORE	Never	12.5	50.0	87.5	0.432
	Rarely	0.0	25.0	50.0	
	Sometimes	0.0	25.0	50.0	
	Almost always	0.0	12.5	50.0	
	Always	0.0	25.0	25.0	
PARTA_SCORE	Never	18.9	76.0	102.4	0.020
	Rarely	29.9	59.1	75.2	
	Sometimes	36.0	49.4	55.5	
	Almost always	22.5	33.9	59.1	
	Always	24.7	40.2	62.8	
SS_SCORE	Never	7.4	24.4	48.9	0.002
	Rarely	17.6	20.6	45.6	
	Sometimes	14.7	20.6	33.8	
	Almost always	8.8	22.1	29.4	
	Always	8.8	16.7	20.6	
qol_fisSCORE	Never	14.1	31.3	48.4	0.292
	Rarely	18.8	25.0	34.4	
	Sometimes	20.8	31.3	37.5	
	Almost always	17.2	25.0	31.3	
	Always	12.5	25.0	31.3	
qol_funSCORE	Never	1.4	8.3	27.8	0.004
	Rarely	8.3	11.1	44.4	
	Sometimes	16.7	33.3	44.4	
	Almost always	11.1	22.2	44.4	
	Always	11.1	33.3	44.4	
qol_socSCORE	Never	7.8	12.5	31.3	0.057
	Rarely	0.0	12.5	34.4	
	Sometimes	12.5	25.0	35.4	
	Almost always	12.5	25.0	43.8	
	Always	6.3	25.0	43.8	
qol_sesSCORE	Rarely	4.2	29.2	50.0	0.018

	Sometimes	0.0	25.0	50.0	
	Almost always	20.8	33.3	50.0	
	Always	25.0	41.7	50.0	
qol_fatSCORE	Never	17.3	28.6	41.7	<0.001
	Rarely	26.2	31.0	40.5	
	Sometimes	33.3	35.7	41.1	
	Almost always	28.6	35.7	42.0	
	Always	35.7	40.5	45.2	
qolgenv_SCORE	Never	11.6	21.8	34.2	0.030
	Rarely	15.4	19.3	39.1	
	Sometimes	25.7	30.8	33.2	
	Almost always	19.1	29.9	37.1	
	Always	21.9	27.2	39.6	
qol_SPESCORE	Never	11.9	19.9	21.9	<0.001
	Rarely	10.1	17.9	40.5	
	Sometimes	19.0	26.2	34.5	
	Almost always	16.7	31.0	42.0	
	Always	23.8	34.7	44.0	
qolall_SCORE	Never	18.1	26.2	34.8	0.019
	Rarely	13.3	21.4	38.5	
	Sometimes	21.4	27.8	34.3	
	Almost always	21.9	30.0	38.4	
	Always	23.1	32.1	40.9	
qolallv_SCORE	Never	11.7	21.4	31.7	0.002
	Rarely	14.7	17.4	39.1	
	Sometimes	25.0	29.1	32.2	
	Almost always	20.3	29.1	38.8	
	Always	22.7	29.3	39.5	
qol_toiSCORE	Never	10.0	18.9	32.7	0.003
	Rarely	11.9	18.0	38.7	
	Sometimes	23.4	27.6	32.8	
	Almost always	17.7	26.2	35.6	
	Always	19.8	27.8	38.3	

Values are weighted means (definition 1)

HM-PRO, Hematological Malignancy-Patient Reported Outcomes; QOL-E, psychometric questionnaire assessing HRQoL in MDS patients. QoL, quality of life; QOL-ALL, calculated by taking the mean of QOL-GEN and QOL-MDSS; QOL-FAT, fatigue; QOL-FIS, physical well-being; QOL-FUN, functional well-being; QOL-GEN, calculated by taking the mean of all domains except for QOL-MDSS; QOL-SEX, sexual well-being; QOL-SOC, social/family well-being; QOL-MDSS, MDS-specific disturbances; QOL-TOI, treatment outcome index calculated by taking the mean of QOL-FIS, QOL-FUN, and QOL-MDSS

Supplementary Table S2. QOL-E and HM-PRO scores according to negative impact of treatment on everyday life

**Negative impact of treatment on everyday life?**		**Percentiles**			
		**25**	**Median**	**75**	**p value**
PB_SCORE	Not at all	3.6	28.6	53.6	<0.001
	A little	35.7	57.1	72.3	
	A lot	46.4	64.3	82.1	
EB_SCORE	Not at all	13.6	36.4	45.5	<0.001
	A little	22.7	50.0	60.2	
	A lot	47.7	59.1	72.7	
SW_SCORE	Not at all	0.0	50.0	6.7	0.004
	A little	29.2	6.7	83.3	
	A lot	41.7	83.3	108.3	
ED_SCORE	Not at all	0.0	0.0	25.0	<0.001
	A little	0.0	25.0	50.0	
	A lot	0.0	25.0	75.0	
PARTA_SCORE	Not at all	14.5	34.4	52.4	<0.001
	A little	30.0	53.1	63.4	
	A lot	41.2	50.5	85.8	
SS_SCORE	Not at all	8.8	17.6	20.6	<0.001
	A little	16.9	26.6	40.8	
	A lot	22.1	32.4	47.1	
qol_fisSCORE	Not at all	15.6	31.3	37.5	<0.001
	A little	15.6	25.0	31.3	
	A lot	18.8	25.0	34.4	
qol_funSCORE	Not at all	20.8	44.4	50.0	<0.001
	A little	11.1	16.7	44.4	
	A lot	8.3	11.1	22.2	
qol_socSCORE	Not at all	12.5	37.5	50.0	<0.001
	A little	10.9	21.9	31.8	
	A lot	0.0	6.3	18.8	
qol_sesSCORE	Not at all	25.0	50.0	50.0	<0.001
	A little	15.6	25.0	50.0	
	A lot	0.0	8.3	27.1	
qol_fatSCORE	Not at all	34.5	40.5	45.2	<0.001
	A little	28.6	35.7	38.7	
	A lot	28.6	31.0	34.5	
qolgenv_SCORE	Not at all	24.9	32.3	42.4	<0.001
	A little	19.1	23.2	33.2	
	A lot	16.4	19.3	25.1	
qol_SPESCORE	Not at all	30.4	37.5	45.8	<0.001
	A little	13.3	22.0	30.7	
	A lot	9.5	17.9	20.2	
qolall_SCORE	Not at all	29.8	33.9	41.7	<0.001
	A little	20.0	24.3	30.3	
	A lot	13.3	19.3	23.0	
qolallv_SCORE	Not at all	28.2	32.0	43.1	<0.001
	A little	17.9	24.7	32.4	
	A lot	15.0	17.5	24.0	
qol_toiSCORE	Not at all	25.8	33.6	42.4	<0.001
	A little	15.6	20.5	32.4	
	A lot	14.6	18.9	23.5	

HM-PRO, Hematological Malignancy-Patient Reported Outcomes; QOL-E, psychometric questionnaire assessing HRQoL in MDS patients. QoL, quality of life; QOL-ALL, calculated by taking the mean of QOL-GEN and QOL-MDSS; QOL-FAT, fatigue; QOL-FIS, physical well-being; QOL-FUN, functional well-being; QOL-GEN, calculated by taking the mean of all domains except for QOL-MDSS; QOL-SEX, sexual well-being; QOL-SOC, social/family well-being; QOL-MDSS, MDS-specific disturbances; QOL-TOI, treatment outcome index calculated by taking the mean of QOL-FIS, QOL-FUN, and QOL-MDSS

Supplementary Table S3. QOL-E and HM-PRO scores according to patients feeling to be a burden for their family

**I feel I am a burden**		**Percentiles**			
**to my family**		**25**	**Median**	**75**	**p value**
PB_SCORE	Yes	46.4	64.3	77.7	
	Not sure	42.6	50.0	71.4	
	No	7.1	29.3	59.3	<0.001
EB_SCORE	Yes	44.3	59.1	67.0	
	Not sure	34.1	56.8	73.9	
	No	13.6	31.8	48.9	<0.001
SW_SCORE	Yes	50.0	6.7	100.0	
	Not sure	45.8	6.7	83.3	
	No	0.0	41.7	6.7	<0.001
ED_SCORE	Yes	6.3	37.5	50.0	
	Not sure	0.0	25.0	50.0	
	No	0.0	0.0	25.0	<0.001
PARTA_SCORE	Yes	44.0	53.3	71.0	
	Not sure	40.0	52.1	62.9	
	No	20.4	29.4	54.2	<0.001
SS_SCORE	Yes	18.4	26.5	31.6	
	Not sure	20.6	30.9	45.6	
	No	8.8	17.6	26.5	<0.001
qol_fisSCORE	Yes	14.1	21.9	29.7	
	Not sure	17.2	25.0	32.8	
	No	17.2	31.3	36.6	<0.001
qol_funSCORE	Yes	11.1	16.7	25.0	
	Not sure	11.1	20.8	42.4	
	No	11.1	33.3	50.0	<0.001
qol_socSCORE	Yes	0.0	0.0	6.3	
	Not sure	6.3	15.6	26.6	
	No	25.0	35.4	50.0	<0.001
qol_sesSCORE	Yes	0.0	25.0	44.8	
	Not sure	17.7	25.0	50.0	
	No	25.0	41.7	50.0	<0.001
qol_fatSCORE	Yes	28.6	31.0	37.5	
	Not sure	28.6	33.3	40.5	
	No	33.3	38.1	44.6	<0.001
qolgenv_SCORE	Yes	16.0	18.8	21.4	
	Not sure	19.1	24.5	31.9	
	No	23.7	31.9	41.7	<0.001
qol_SPESCORE	Yes	9.8	17.9	25.0	
	Not sure	18.8	26.6	31.3	
	No	21.1	34.5	43.0	<0.001
qolall_SCORE	Yes	13.4	19.7	24.1	
	Not sure	21.7	26.5	30.8	

	No	25.0	33.5	41.2	0.002
qolallv_SCORE	Yes	14.4	17.8	21.6	
	Not sure	20.2	25.9	29.8	
	No	24.8	32.4	42.3	<0.001
qol_toiSCORE	Yes	14.0	18.8	23.7	
	Not sure	16.7	26.4	33.2	
	No	20.4	29.8	41.5	<0.001

Values are weighted means (definition 1)

HM-PRO, Hematological Malignancy-Patient Reported Outcomes; QOL-E, psychometric questionnaire assessing HRQoL in MDS patients. QoL, quality of life; QOL-ALL, calculated by taking the mean of QOL-GEN and QOL-MDSS; QOL-FAT, fatigue; QOL-FIS, physical well-being; QOL-FUN, functional well-being; QOL-GEN, calculated by taking the mean of all domains except for QOL-MDSS; QOL-SEX, sexual well-being; QOL-SOC, social/family well-being; QOL-MDSS, MDS-specific disturbances; QOL-TOI, treatment outcome index calculated by taking the mean of QOL-FIS, QOL-FUN, and QOL-MDSS
